# Endemic carbapenem-nonsusceptible *Acinetobacter baumannii-calcoaceticus* complex in intensive care units of the national referral hospital in Jakarta, Indonesia

**DOI:** 10.1186/s13756-017-0296-7

**Published:** 2018-01-12

**Authors:** Yulia Rosa Saharman, Anis Karuniawati, Rudyanto Sedono, Dita Aditianingsih, Pratiwi Sudarmono, Wil H. F. Goessens, Corné H. W. Klaassen, Henri A. Verbrugh, Juliëtte A. Severin

**Affiliations:** 1 Department of Clinical Microbiology, Faculty of Medicine, Universitas Indonesia/ Dr. Cipto Mangunkusumo General Hospital, Jakarta, Indonesia; 2 Department of Medical Microbiology and Infectious Diseases, Erasmus MC University Medical Center Rotterdam, ‘s-Gravendijkwal 230, 3015 CE Rotterdam, The Netherlands; 3 Critical Care Division, Department of Anesthesia and Intensive Care, Faculty of Medicine, Universitas Indonesia / Dr. Cipto Mangunkusumo General Hospital, Jakarta, Indonesia

**Keywords:** *Acinetobacter baumannii-calcoaceticus* complex, Intensive care unit, Carbapenems, Antimicrobial resistance, Carbapenemase, Indonesia

## Abstract

**Background:**

Carbapenem-nonsusceptible *A. baumannii-calcoaceticus* complex have emerged worldwide, but the epidemiology in Indonesian hospitals has not been studied.

**Methods:**

A prospective observational study was performed on the intensive care units (ICUs) of the national referral hospital in Jakarta-Indonesia, in 2013 and 2014. All consecutive adult patients admitted and hospitalized for >48 h in ICUs were included. Basic and clinical data at admission were recorded. Carbapenem-nonsusceptible *A. baumannii-calcoaceticus* complex from clinical cultures and standardized screening were included. Environmental niches and healthcare workers (HCWs) were also screened. PCR was used to detect carbapenemase genes, and Raman spectroscopy as well as multilocus sequence typing (MLST) for typing.

**Results:**

Of 412 included patients, 69 (16.7%) carried carbapenem-nonsusceptible *A. baumannii-calcoaceticus* complex on admission, and 89 (25.9%) became positive during ICU stay. The acquisition rate was 43 per 1000 patient-days at risk. Six isolates were cultured from environment and one from a HCW. Acquisition of carbapenem-nonsusceptible *A. baumannii-calcoaceticus* complex was associated with longer ICU stay (median interquartile range [IQR]: 11 days [5–18], adjusted hazard ratio [aHR]: 2.56 [99% confidence interval (CI):1.76–3.70]), but not with mortality (adjusted odds ratio: 1.59 [99%CI: 0.74–3.40] at the chosen level of significance). The *bla*_OXA-23_-like gene was detected in 292/318 (91.8%) isolates, including isolates from the environment and HCW. Typing revealed five major clusters. Sequence types (ST)195, ST208, ST218, ST642 as well as new STs were found. The dominant clone consisted of isolates from patients and environment throughout the study period.

**Conclusions:**

Carbapenem-nonsusceptible *A. baumannii-calcoaceticus* complex are endemic in this setting. Prevention requires source control and limiting transmission of strains.

**Trial registration:**

The study was retrospectively registered at www.trialregister.nl (No:5541). Candidate number: 23,527, NTR number: NTR5541, Date registered NTR: 22nd December 2015.

**Electronic supplementary material:**

The online version of this article (10.1186/s13756-017-0296-7) contains supplementary material, which is available to authorized users.

## Background

Multidrug-resistant *Acinetobacter baumannii-calcoaceticus* complex has emerged as one of the most problematic pathogens in hospitals. Their natural habitat is in the environment, including niches in the hospital from which they can spread to patients [1]. Risk factors for colonization and infection with multidrug-resistant *A. baumannii-calcoaceticus* complex include length of hospital stay, admission to an intensive care unit (ICU), mechanical ventilation, antimicrobial exposure, and several other factors [2]. Carbapenem-nonsusceptible *A. baumannii-calcoaceticus* complex is considered a significant health problem because of the limited options remaining for antibiotic treatment [3].

In 2013, the Centers for Disease Control and Prevention of the United States reported an estimated 12,000 healthcare-associated *Acinetobacter* infections. Nearly 7000 of these were caused by multidrug-resistant isolates [4]. In 2008, Lagamayo et al. reported that between 2 and 77% of all clinical isolates of *Acinetobacter* spp. in Asian countries were resistant to imipenem, and that multidrug-resistant *Acinetobacter* spp. were highly prevalent, particularly in Thailand and India, but not in the Philippines [5].

To date, there have been no data on the epidemiology of carbapenem-resistant or -nonsusceptible *A. baumannii-calcoaceticus* complex from Indonesia, the fourth most populous country in the world. This study was designed to delineate the clinical and molecular epidemiology of carbapenem-nonsusceptible *A. baumannii-calcoaceticus* complex in two ICUs of the national referral hospital in Jakarta, Indonesia.

## Methods

### Study design

A prospective observational study was performed at the Dr. Cipto Mangunkusumo General Hospital, a 1000-bed teaching hospital in Jakarta, Indonesia, from April–October 2013 and from April–August 2014. We conducted this study in two ICUs: the 12-bedded adult ICU and the 8-bedded Emergency Room (ER)-ICU with an average of 1010 and 415 admissions per year, respectively. The adult ICU is an open ward with mechanical ventilation facilities, admitting patients with mixed medical and surgical indications, and one designated nurse per patient during first shifts (7 am-3 pm) and a 1:1.5 nurse/patient ratio during other shifts. The ER-ICU has the same design, and the nurse-to-patient ratio in the first shifts is 1:1 and during the other shifts 1:2. The populations served by these two ICUs were identical, and there was also no difference in the service provided.

The study was performed in the framework of a larger study that focused on carbapenem-nonsusceptible *Klebsiella pneumoniae*, *Pseudomonas aeruginosa*, and *A. baumannii-calcoaceticus* complex.

All adult patients (≥18 years old) admitted to one of the two ICUs and hospitalized for more than 48 h were eligible for enrollment in this study. The first screening cultures were taken on the day of admission, and if a patient was discharged before 48 h, he or she was excluded. Informed consent was obtained from the patient or their relatives as applicable. Demographic and clinical characteristics such as age, gender, medical or surgical indication, underlying diseases, hospitalization history, and previous use of antibiotics were recorded on admission.

Systemic inflammatory response syndrome (SIRS) criteria on admission were used as a screening tool to assess (severity of) septic illness. The SIRS criteria were calculated and included in the study, as this was practice at the time of the study [6].

The quick Sequential Organ Failure Assessment (qSOFA) score is a new bedside prompt that may identify patients with suspected infection and helps to determine sepsis in all healthcare environments. The qSOFA score assigns one point for each of the following conditions: systolic blood pressure ≤ 100 mmHg, respiratory rate ≥ 22 breaths per minute, and altered mentation (Glasgow coma scale <15). A qSOFA score ≥ 2 at the onset of infection is associated with a greater risk of death and prolonged ICU stay [6].

The primary outcome measure was acquisition of a carbapenem-nonsusceptible *A. baumannii-calcoaceticus* complex. Acquisition is defined as a screening culture or clinical culture with first detection of *A. baumannii-calcoaceticus* complex, with reduced susceptibility to a carbapenem, that was not present within the first 48 h of admission. Secondary outcome measures were length of stay in the ICU, and mortality during ICU stay.

Environmental samples (Additional file 1: Table S1), were taken twice (in October 2013 and December 2014), simultaneously in both ICUs. Screening of healthcare workers (HCWs) was performed once. HCWs were defined as all personnel including doctors, nurses and other people (cleaning staff, administration staff, porters, nutritionist) working in one of the two ICUs during the study period.

### Screening method

From patients enrolled, screening cultures were obtained from throat and rectum or stools by experienced ICU nurses who had been trained for the task of taking the samples, on the day of admission, at the time of discharge from the ICU, and weekly if the patient was admitted for seven days or more. Sampling was performed using sterile cotton-tipped swabs, and swabs were transported to the laboratory in Amies transport medium (Oxoid, Basingstoke, UK). The swabs in medium were transported in clean, closed boxes at ambient temperature to the laboratory on the same day. All swabs were processed in the laboratory within 24 h.

Clinical samples were collected on indication from patients under aseptic precautions from the lower respiratory tract, blood, urine, tissue, or wound.

Environmental samples were taken from various sites, including washbasins, bed rails, bedside cabinet tables, ventilators, and monitor screens (Additional file 1: Table S1), with sterile cotton-tipped swabs and placed in Amies transport medium.

All HCWs working in one of the ICUs were sampled once over the course of one month (September 2013) with sterile cotton-tipped swabs, which were transported to the laboratory in Amies transport medium.

### Microbiological methods

#### Isolation and identification

In the Clinical Microbiology Laboratory of Faculty of Medicine, Universitas Indonesia, Jakarta, each swab was placed in 5 ml trypticase soy broth (TSB) supplemented with cefotaxime 2 mg/L plus vancomycin 50 mg/L and incubated overnight. The next day, a loop of broth (10 μl) was subsequently subcultured onto MacConkey agar (Oxoid) and incubated aerobically at 37 °C for 16–24 h, following which identification using the VITEK2® system (bioMérieux, Lyon, France) and susceptibility testing of colonies suggestive of *A. baumannii-calcoaceticus* complex was performed. All swabs, i.e. from patients, healthcare workers (HCWs), and environmental screening were processed in the same way.

Blood cultures were collected in BACTEC® (BD, Franklin Lakes, NJ, USA) bottles as per manufacturer’s instructions at the discretion of attending clinicians with a minimum of 10 ml of blood collected from at least two puncture sites. Other clinical specimens were inoculated onto blood and MacConkey agar plates and incubated for 24 h at 37 °C. Subsequently, all colonies that had been cultured were examined for morphology by Gram stain and identified using the VITEK2® system.

Strains were stored in duplicate in −80 °C in TSB with glycerol 10%. One tube of each strain was sent to the Department of Medical Microbiology and Infectious Diseases, Erasmus MC, Rotterdam, the Netherlands, which laboratory holds an ISO 15189 accreditation, for further analysis. The other tube of each strain remained in the Indonesian laboratory. In the Netherlands, the identity of strains was confirmed using matrix-assisted laser desorption/ionisation (Maldi Biotyper, Bruker Microflex LT, London, UK).

The quality control strains used for this part of the study in Indonesia were *Escherichia coli* ATCC 25922 and *Pseudomonas aeruginosa* ATCC 27853, in the laboratory in Erasmus MC multiple quality control strains were used.

#### Antimicrobial susceptibility testing

The susceptibility of the screening isolates to imipenem and meropenem was investigated by standard Kirby-Bauer disc diffusion technique using Mueller-Hinton agar plates (BD). For the isolates from clinical cultures, approximation of the minimum inhibitory concentrations (MICs) of antibiotics was determined by the VITEK2® system. Carbapenem zone sizes and MICs were interpreted according to EUCAST (2013) using the following breakpoints: meropenem zone size <21 mm and MIC >2 mg/L, imipenem zone size <23 mm and MIC >2 mg/L. [7] For this part of the study, quality control strains as described above were used.

#### DNA extraction and carbapenemase gene detection

DNA from the isolates was extracted by a cell lysis step and boiling using the InstaGene Matrix (Bio-Rad Laboratories, USA) according to the manufacturer’s instructions. PCR-based detection of Ambler class B metallo-beta-lactamases (*bla*_NDM_), class D beta-lactamases (*bla*_OXA-23-like_, *bla*_OXA-24-like_, *bla*_OXA-51-like_ and *bla*_OXA-58-like_) and IS*Aba1* were carried out using a T3000 Thermocycler (Biometra-Whatman, Goettingen). The upstream location of the IS*Aba1* insertion element of the *bla*_OXA__-23-like_ gene was demonstrated by using the IS*Aba1* forward primer and the *bla*_OXA-23-like_ reverse primer. PCR primers and reaction conditions for PCR were as described previously [8–11]. Amplified PCR products were resolved by electrophoresis at 250 V for 30 min on 1.5% agarose gels with 0.5 x Tris (89 mM)-boric acid (89 mM)-EDTA(2 mM) buffer containing SyBr® Safe DNA Gel Stain and visualized under UV light and photographed. In each run, a positive and negative control was included.

#### Clonal relatedness

Raman spectroscopy (SpectraCell RA® Bacterial Strain Analyzer, RiverD International BV, Rotterdam, The Netherlands) was applied as a first typing method [12, 13]. All isolates were grown overnight on trypticase soy agar (TSA; BD). Samples were prepared and submitted to spectrometry as described previously [13]. Raman light scatterings were analyzed by SpectraCell*RA* software version 1.9.0.13444:24. The similarity between pairs of spectra was calculated using the squared Pearson correlation coefficient (R^2^-values), multiplied by 100 and expressed as a percentage. The similarity threshold for this study was set at 91% so that two isolates with an R^2^ below this threshold were considered to be different and were designated different Raman types. Two isolates with an R^2^-value between 91% and 100% were considered indistinguishable and were considered to have the same Raman type. Correlation matrices displayed as 2D plots diagram were created using MATLAB version 7.1 (The MathWorks, Natick, MA, USA).

Multilocus sequence typing (MLST) was used as a second typing method for a subset of isolates, including isolates from the largest clones of Raman spectroscopy, and all isolates from blood cultures (one per patient). These isolates were subjected to whole genome sequencing (WGS) using Illumina chemistry. MLST typing results were deduced from the WGS data and assigned based on the Oxford database (pubmlst.org/abaumannii).

### Statistical analysis

Statistical analyses were done using SPSS Version 24.0 (SPSS, Chicago, IL, USA). Patients admitted to adult ICU were compared to ER-ICU using Chi square or Fisher’s Exact and Mann-Whitney as appropriate. One-way ANOVA was used to compare patient characteristics according to their *A. baumannii-calcoaceticus* complex status. Univariate and multivariate analyses were performed to establish risk factors associated with mortality using a multivariate logistic regression model with backward selection and inclusion of variables with a *p*-value <0.1 in the univariate analysis. Cox proportional regression was used to analyse risk factors for length of stay. Kaplan-Meier method was performed to construct survival curves. *P*-values of less than 0.01 were considered significant [14].

## Results

### Patient characteristics

During the 11-month study period, 1211 patients were hospitalized in the ICUs (Adult ICU: 863, ER-ICU: 348). Additional file 1: Table S2 shows baseline characteristics of patients in each ICU. Of the 412 included patients, 188 were admitted to the adult ICU and 224 to the ER-ICU. There were no significant differences in characteristics between patients in both ICUs, except that in the adult ICU most of the patients had been referred from another ward in the same hospital (Additional file 1: Table S2). Therefore, we analyzed the data from the ICUs both separately and pooled.

**Fig. 1:**
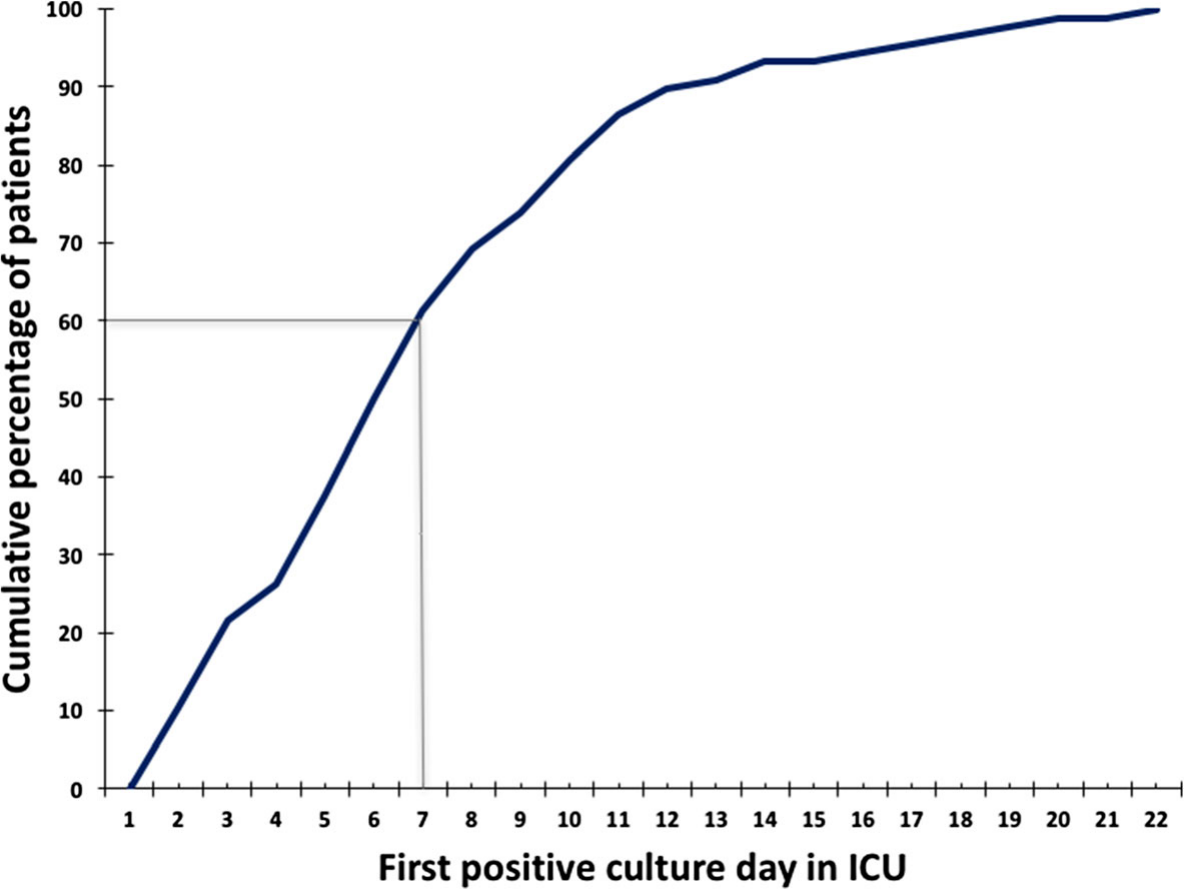
Acquisition of carbapenem-nonsusceptible *Acinetobacter baumannii-calcoaceticus* complex in ICUs. Note: The solid line represents the cumulative percentage of patients by first day of culture being positive for carbapenem-nonsusceptible *A. baumannii-calcoaceticus* complex during ICU stay. In total, data from 89 patients are included in this figure. The median acquisition day (day 7, 60% of patients positive) is shown as well

Overall, 158/412 (38.3%) patients had a positive culture with carbapenem-nonsusceptible *A. baumannii-calcoaceticus* complex, the remaining 254 patients were free from carbapenem-nonsusceptible *A. baumannii-calcoaceticus* complex on admission and remained so during their ICU stay. Sixty-nine patients (69/412; 16.7%) already carried carbapenem-nonsusceptible *A. baumannii-calcoaceticus* complex as revealed by screening cultures taken on the day of ICU admission, 89/343 (25.9%) patients who were initially culture-negative acquired carbapenem-nonsusceptible *A. baumannii-calcoaceticus* complex during their ICU stay (Additional file 2: Figure S1). Of the total of 158 patients with positive cultures, the positive cultures were obtained from screening specimens only in 80 patients, from clinical specimens only in 34 patients and from both screening and clinical samples in 44 patients. Interestingly, of the patients that were positive on ICU admission, 17 (24.6%) were admitted directly from the emergency unit. Six patients had one or more blood cultures with carbapenem-nonsusceptible *A. baumannii-calcoaceticus* complex, and three of them died on the ICU. The dynamics of acquisition of carbapenem-nonsusceptible *A. baumannii-calcoaceticus* complex in the ICU is shown in Fig. 1, 60% of patients that became positive for carbapenem-nonsusceptible *A. baumannii-calcoaceticus* complex during their ICU stay did so in the first week of ICU stay. There were no differences in the dynamics of carbapenem-nonsusceptible *A. baumannii-calcoaceticus* complex acquisition between the two ICUs (median acquisition day in adult ICU: 7, in ER-ICU: 6). The acquisition rate for carbapenem-nonsusceptible *A. baumanni-calcoaceticus* complex was 43 per 1000 patient-days at risk overall, with an average of 43 per 1000 patient-days in the adult ICU and 43 per 1000 patient-days in the ER-ICU.

**Fig. 2:**
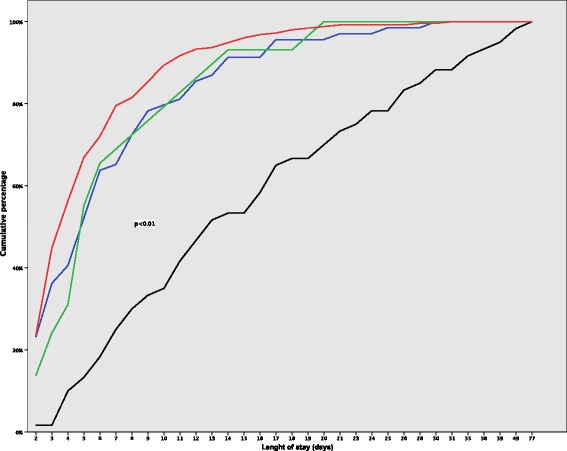
Cumulative percentage of length of stay for patients according to their carbapenem-nonsusceptible *Acinetobacter baumannii-calcoaceticus* complex status. Note: Lengths of stay (days) represent total days patients were hospitalized in the ICU. The red line represents patients that were always carbapenem-nonsusceptible *A. baumannii****-****calcoaceticus* complex negative during their ICU stay. The blue line represents patients already carbapenem-nonsusceptible *A. baumannii****-****calcoaceticus* complex positive on the day of admission. The green line represents patients that were carbapenem-nonsusceptible *A. baumannii****-****calcoaceticus* complex positive only at the time of discharge and the black line represents patients that became positive for carbapenem-nonsusceptible *A. baumannii****-****calcoaceticus* complex during their ICU stay before the day of discharge. *P* value: comparison between patients that became positive with carbapenem-nonsusceptible *A. baumannii****-****calcoaceticus* complex before the day of discharge and the other groups

Patient outcomes were clearly associated with carbapenem-nonsusceptible *A. baumanni-calcoaceticus* complex status of patients. Patients who acquired carbapenem-nonsusceptible *A. baumannii-calcoaceticus* complex during their ICU stay had a significantly longer length of stay (median [interquartile range (IQR)]: 11 [5–18], adjusted hazard ratio [aHR]: 2.56 [99% confidence interval (CI): 1.76–3.70], *p* < 0.001, Additional file 1: Table S4, particularly the group of patients that became positive before the day of their discharge (median [IQR] 13 [8–23] days, *p* < 0.001, Fig. 2) compared to the other groups of patients, of which ≥80% were discharged from the ICU within ten days. Interestingly, these latter groups not only included the patients that were always free from carbapenem-nonsusceptible *A. baumannii-calcoaceticus* complex, but also included patients that already carried carbapenem-nonsusceptible *A. baumannii-calcoaceticus* complex at the time of admission to the ICU, and patients that remained free of carbapenem-nonsusceptible *A. baumannii-calcoaceticus* complex until they were found to be positive by screening on the day of their discharge from the ICU (Fig. 2).

**Fig. 3:**
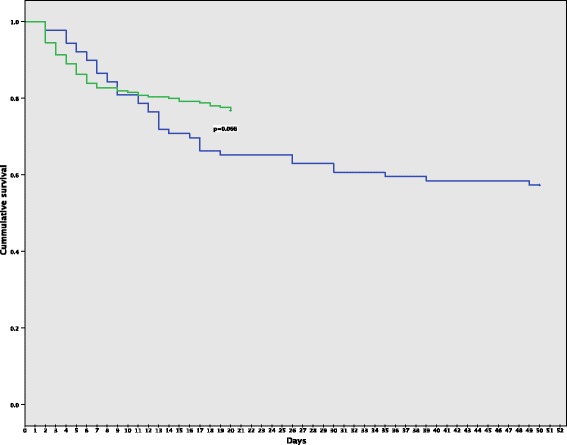
Survival analysis of ICU patients according to their carbapenem-nonsusceptible *Acinetobacter baumannii-calcoaceticus* complex status. Note: Survival of patients with carbapenem-nonsusceptible *A. baumannii****-****calcoaceticus* complex acquired during their ICU stay (blue line) compared with the survival of patients that remained negative for carbapenem-nonsusceptible *A. baumannii****-****calcoaceticus* complex in their screening and clinical cultures (green line)

Acquisition of carbapenem-nonsusceptible *A. baumannii-calcoaceticus* complex was not associated with mortality, 23.2% of patients that remained free of carbapenem-nonsusceptible *A. baumannii-calcoaceticus* complex died versus 42.7% of patients that acquired carbapenem-nonsusceptible *A. baumannii-calcoaceticus* complex during their ICU stay (Fig. 3, *p* = 0.066; Additional file 1: Table S3, multivariate analysis: adjusted Odds Ratio (aOR):1.59 [99%CI: 0.74–3.40]). Importantly, the admission SIRS and qSOFA scores of patients with or without *A. baumannii-calcoaceticus* complex acquisition did not differ (Table 1), indicating that the difference in the risk of dying was not present at the time of ICU admission but emerged later during their ICU stay (SIRS: crude Odds Ratio (cOR):1.69 [99%CI:0.55–5.22], *p* = 0.230; qSOFA:cOR: 1.45[99%CI:0.68–3.08], *p* = 0.211, Additional file 1: Table S3).

**Table 1:** Patient characteristics and outcomes according to carbapenem-nonsusceptible *A. baumannii****-****calcoaceticus* complex status

	Carbapenem-nonsusceptible *A. baumannii****-****calcoaceticus* complex positive on admission	Carbapenem-nonsusceptible *A. baumannii****-****calcoaceticus* complex acquired during ICU stay	Carbapenem-nonsusceptible *A. baumannii****-****calcoaceticus* complex negative	*p* value
	*n* = 69	(*n* = 89)	*n* = 254	
Age (years), median (IQR)	47 (33–60)	48 (35.3–57)	46 (32–58)	0.700
Gender (%)				0.535
Male	35 (50.7)	42 (47.2)	137 (53.9)	
Female	34 (49.3)	47 (52.8)	117 (46.1)	
Underlying diseases (%)				
Cardiovascular				0.024
Yes	9 (13.0)	3 (3.4)	13 (5.1)	
No	60 (87.0)	86 (96.6)	241 (94.9)	
Cerebrovascular				0.001
Yes	3 (4.3)	14 (15.7)	12 (4.7)	
No	66 (95.7)	75 (84.3)	242 (95.3)	
Chronic kidney disease				0.915
Yes	5 (7.2)	8 (9.0)	20 (7.9)	
No	64 (92.8)	81 (91.0)	234 (92.1)	
Diabetes mellitus				0.334
Yes	20 (29.0)	20 (22.5)	78 (30.7)	
No	49 (71.0)	69 (77.5)	176 (69.3)	
Malignancy				0.740
Yes	29 (42.0)	37 (41.6)	116 (45.7)	
No	40 (58.0)	52 (58.4)	138 (54.3)	
Indication for ICU admission (%)				0.002
Medical	32 (46.4)	38 (42.7)	70 (27.6)	
Surgical	37 (53.6)	51 (57.3)	184 (72.4)	
Referral from (%)				0.900
Other ward this hospital	38 (55.1)	48 (53.9)	136 (53.5)	
Other hospital	14 (20.3)	14 (15.7)	49 (19.3)	
Directly from Emergency Unit	17 (24.6)	27 (30.3)	69 (27.2)	
Antibiotic exposure (pre-ICU admission)				
Any antibiotic (%)	58 (84.1)	73 (82.0)	180 (70.9)	0.021
Carbapenem (%)	24 (34.8)	22 (24.7)	33 (13.0)	<0.01
SIRS Score, (%)				0.916
Score ≥ 2	64 (92.8)	81 (91.0)	232 (91.3)	
Score < 2	5 (7.2)	8 (9.0)	22 (8.7)	
qSOFA Score, (%)				0.089
Score ≥ 2	51 (73.9)	78 (87.6)	205 (80.7)	
Score < 2	18 (26.1)	11 (12.4)	49 (19.3)	
Procedures (during ICU admission)				
Mechanical ventilation (%)	63 (91.3)	88 (98.9)	220 (86.6)	0.004
Mechanical ventilation (days) median(IQR)	5 (2–8)	8 (4–16)	3 (1–6)	
≥ 5 days (%)	36 (52.2)	63 (70.8)	83 (2.7)	<0.01
< 5 days (%)	33 (47.8)	26 (29.2)	171 (67.3)	
Central venous catheter (%)	66 (95.7)	85 (95.5)	212 (83.5)	<0.01
Central venous catheter (days) median(IQR)	6 (3–9)	10 (5–17)	4 (2–7)	
≥ 5 days (%)	41 (59.4)	71 (79.8)	111 (3.7)	<0.01
< 5 days (%)	28 (40.6)	18 (20.2)	143 (56.3)	
Urine catheter	69 (100)	89 (100)	254 (100)	N/A
Urine catheter (days) median (IQR)	6 (3–10)	10 (6–18)	5 (3–7)	
≥ 5 days (%)	26 (37.7)	13 (14.6)	122 (48.0)	<0.01
< 5 days (%)	43 (62.3)	76 (85.4)	132 (52.0)	
Antibiotic therapy (during ICU admission)				
Any antibiotic (%)	68 (98.6)	89 (100)	249 (98.0)	0.411
Carbapenem (%)	42 (60.9)	62 (69.7)	95 (37.4)	<0.01
Outcomes				
Length of stay (days), median (IQR)	5 (3–9)	11 (5–18)	4 (3–7)	<0.01
Death	22 (31.9)	38 (42.7)	59 (23.2)	0.002

Abbreviations: ICU, Intensive Care Unit; IQR, Interquartile range; qSOFA, quick Sepsis-related Organ Failure Assessment; SIRS, Systemic Inflammatory Response Syndrome

Significance was calculated using Oneway ANOVA and Pearson Chi Square

A *p*-value less than 0.01 was considered statistically significant

Patients that were free of carbapenem-nonsusceptible *A. baumannii-calcoaceticus* complex during their entire ICU stay were less likely to have had prior exposure to antibiotics, especially carbapenems (*p* < 0.01), they were more likely to have had a surgical indication for their admission to the ICU, and less likely to have had cerebrovascular disease (Table 1). Patients that acquired carbapenem-nonsusceptible *A. baumannii-calcoaceticus* complex during ICU stay had undergone a procedure (mechanical ventilation), had a medical device (central venous catheter or urine catheter) or had received carbapenem therapy more often than the other groups in the univariate analysis (*p* < 0.01) (Table 1). In a multivariate comparison of patients who acquired carbapenem-nonsusceptible *A. baumannii-calcoaceticus* complex to patients that were always negative, only carbapenem therapy during ICU admission could be identified as a risk factor (aOR: 3.37 [99%CI: 1.68–6.77], *p* < 0.01).

### Carbapenem-nonsusceptible *A*. *baumannii-calcoaceticus *complex and molecular characterization

In total, we collected 311 carbapenem-nonsusceptible isolates from 158 patients, six carbapenem-nonsusceptible *A. baumannii-calcoaceticus* complex isolates cultured from the environment (table, bed rails, sinks, and tapwater), and a single isolate from a healthcare worker (throat) that was carbapenem-nonsusceptible as well (Additional file 1: Table S5).

The *bla*_OXA-23-like_ gene was demonstrated in 292/318 (91.8%) isolates including isolates from patients, the environment and from the healthcare worker. The *bla*_OXA-24-like_ gene was detected in a single isolate. Coexistence of OXA-23 with other oxacillinases and carbapenemases was found: OXA-23/OXA-58 (1 isolate), and OXA-23/NDM-1 (4 isolates)*.* The *bla*_OXA-23_-like gene was always demonstrated in combination with the IS*Aba1* insertion element upstream to the OXA-23 beta-lactamase. The intrinsic *A. baumannii-calcoaceticus* complex gene *bla*_OXA-51_-like was demonstrated in all isolates. In the subset of isolates that were subjected to WGS (*n* = 14), the *bla*_OXA-51_-like gene involved was *bla*_OXA-66_ in 13 isolates and *bla*_OXA-68_ in one isolate (Table 2).

**Table 2:** Results of MLST analyses of 14 carbapenem-nonsusceptible *A. baumannii****-****calcoaceticus* complex isolates

Sample number	ST	gltA	gyrB	gdhB	recA	cpn60	gpi	rpoD	OXA-51 group	OXA-23	Raman cluster
171bl040813	new ST	1	15	3	2	2	164	3	OXA-66	+	CIPTO-31
262bl211013	new ST	1	15	3	2	2	164	3	OXA-66	+	CIPTO-31
275bl101013	new ST	1	15	3	2	2	61	3	OXA-66	+	CIPTO-31
69E–bed-rails-4	new ST	1	15	3	2	2	164	3	OXA-66	+	CIPTO-31
404re030714	195	1	3	3	2	2	96	3	OXA-66	+	CIPTO-48
91EIGD1214	195	1	3	3	2	2	96	3	OXA-66	+	CIPTO-48
156th250713	new allel/ST	1	3	3	2	2	new	3	OXA-66	+	CIPTO-48
206bl020913	new allel/ST	1	3	3	2	2	new	3	OXA-66	+	CIPTO-46
319bl020514	208	1	3	3	2	2	97	3	OXA-66	+	CIPTO-46
207re300813	new ST	1	3	3	2	2	61	3	OXA-66	+	CIPTO-45
422sp170714	new ST	1	3	3	2	2	61	3	OXA-66	+	CIPTO-45
116sp080713	218	1	3	3	2	2	102	3	OXA-66	+	CIPTO-30
176BA150813	218	1	3	3	2	2	102	3	OXA-66	+	CIPTO-30
153bl290713	642	22	15	13	12	4	169	2	OXA-68	+	CIPTO-39

Abbreviations: MLST, Multilocus Sequence Type; ST, Sequence Type

### Clonal relatedness

**Fig. 4:**
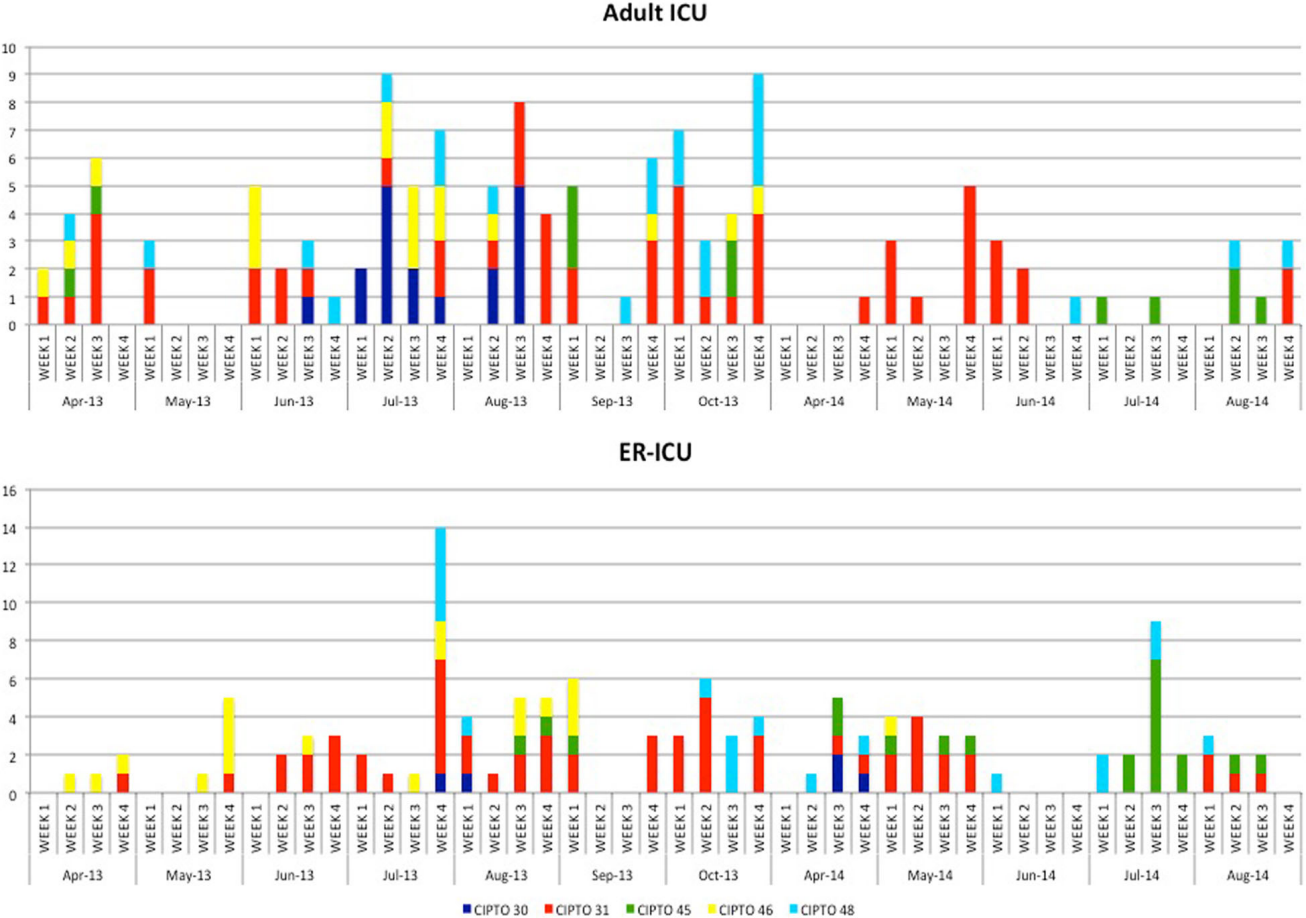
Persistence of Raman clones of carbapenem-nonsusceptible *Acinetobacter baumannii-calcoaceticus* complex in two ICUs of Dr. Cipto Mangunkusomo General Hospital, Jakarta, Indonesia. Note: Endemic curves of the five biggest clusters of carbapenem-nonsusceptible *A. baumannii****-****calcoaceticus* complex in each ICU, April–October 2013 and April–August 2014. The dark blue bars represent cluster CIPTO-30. The red bars represent CIPTO-31. The green bars represent CIPTO-45. The yellow bars represent CIPTO-46 and the light blue bars represent CIPTO-48. The x-axis indicates time of the study (by week). The y-axis indicates number of isolates

Raman spectroscopy analysis performed for all of the isolates, revealed the presence of multiple types within the collection of *A. baumannii-calcoaceticus* complex. In total, 51 Raman types were identified. Interestingly, the majority of strains belonged to one of five major clusters (Additional file 3: Figure S2). The largest cluster (designated CIPTO-31) consisted of 111 isolates obtained from 69 patients (screening and clinical specimens) and four isolates from the environment. The sources of the five major clusters are specified in Additional file 1: Table S6. Strains belonging to the dominant cluster CIPTO-31 were present in both ICUs throughout the study period, whereas other clones seemed to wax and wane over time (Fig. 4). Patients were colonized with carbapenem-nonsusceptible *A. baumannii-calcoaceticus* complex irrespective of the location of their bed in these ICUs indicating that spreading of carbapenem-nonsusceptible *A. baumannii-calcoaceticus* complex in the ICUs was not restricted to only a part of the ICU (Additional file 4: Figure S3).

MLST, performed for a subset of 14 isolates, revealed the presence of multiple sequence types (STs), which corresponded closely to the Raman spectroscopy clustering (Table 2). Four previously identified STs (ST195, ST208, ST218, and ST642) as well as several new STs, and a new allele for the *gpi* gene were found in this study (Table 2).

## Discussion

This is the first report of a study on the clinical and molecular epidemiology of carbapenem-nonsusceptible *A. baumannii-calcoaceticus* complex in two ICUs in a large academic hospital in Jakarta, Indonesia. These two ICUs can be considered to have endemic carbapenem-nonsusceptible strains belonging to *A. baumannii-calcoaceticus* complex, i.e. entrenched by a few carbapenem-nonsusceptible clones, whose acquisition by patients may be associated with a prolonged ICU stay.

Carbapenem-nonsusceptible *A. baumannii-calcoaceticus* complex has emerged globally as a hospital-acquired pathogen, causing many outbreaks, especially in ICUs [3]. In Asia, carbapenem-resistant *A. baumannii-calcoaceticus* complex were found to dominate in Vietnam [15], Thailand [16], Malaysia [17], and also China [18]. Similar to these studies, we found that 38.3% of the patients had colonization or infection with carbapenem-nonsusceptible *A. baumannii-calcoaceticus* complex. By screening on ICU admission, 43.7% of the carbapenem-nonsusceptible *A. baumannii-calcoaceticus* complex positive patients were already colonized with this species prior to their admission. This suggests that patients may become colonized with such strains elsewhere in the same hospital or in another hospital from which they are referred, or may come with such strain directly from the community, possibly having acquired their strain during a previous healthcare contact. Thus, the ICUs in this study experience a regular influx of patients carrying carbapenem-nonsusceptible *A. baumannii-calcoaceticus* complex strains into their setting. Our findings also raise questions about carriage of *A. baumannii-calcoaceticus* complex in the community, a finding that was also reported in a recent study from Semarang, Central Java, Indonesia. From the nasopharynx of 14 healthy people, *A. baumannii-calcoaceticus* complex was isolated in that study [19]. This requires further investigation.

Screening cultures can, therefore, be considered very helpful for early detection, infection control, and rational antibiotic use. A study in South Florida found that patients with positive surveillance cultures had a 8.4-fold higher risk of developing a subsequent *A. baumannii-calcoaceticus* complex infection compared with patients who remained negative on surveillance cultures [20].

Our data also show that many patients acquire carbapenem-nonsusceptible *A. baumannii-calcoaceticus* complex during their ICU stay and that these acquisitions are associated with significantly longer ICU stay but not with mortality (at the chosen level of significance) compared to patients who did not acquire carbapenem-nonsusceptible *A. baumannii-calcoaceticus* while in the ICU. This is in agreement with a study from the USA, which showed an independent association between multidrug-resistant *A. baumannii-calcoaceticus* complex and increased hospital and ICU length of stay, but not an increased mortality [21]. However, a recent systematic review and meta-analysis to examine the association between carbapenem-resistant *A. baumannii-calcoaceticus* complex (CRAB) and mortality found that patients with CRAB had a significantly higher risk of mortality than patients with carbapenem-susceptible *A. baumannii-calcoaceticus* complex (crude OR = 2.22; 95% CI = 1.66–2.98) [22].

The most prevalent mechanisms of carbapenem-nonsusceptibility in *A. baumannii-calcoaceticus* complex are acquired OXA-type carbapenem-hydrolyzing beta-lactamases of the OXA-23, OXA-24 and OXA-58 subfamilies, and the New Delhi metallo-beta-lactamases (NDM) [23–25]. Our study found that 91.8% of the isolates carried the *bla*_OXA-23_-like gene in combination with the upstream presence of the IS*Aba1* insertion element, enhancing carbapenem resistance. *bla*_OXA-24_-like, *bla*_OXA-58_-like, and *bla*_NDM_-like genes were rarely present. The dissemination of OXA-23 producing carbapenem-nonsusceptible *A. baumannii-calcoaceticus* complex isolates has previously been reported in Asia and throughout the world [26–28].

Carbapenem-nonsusceptible *A. baumannii-calcoaceticus* complex colonizing/infecting ICU patients may originate from the patient her/himself, but may also come from contaminated hospital equipment and environment, staff and other patients. Multiple reported outbreaks of multidrug-resistant *A. baumannii-calcoaceticus* complex infection were associated with environmental contamination [29–31]. There should be a focus on the prevention of nosocomial transmission of these microorganisms from these environmental sources to patients.

We performed Raman spectroscopy as a first bacterial typing method [12]. This analysis revealed five clusters, with the largest one (CIPTO-31), responsible for more than one third of all isolates, persisting in both ICUs throughout the study period. Geographical analysis of cluster CIPTO-31 isolates showed spreading of this clone throughout both ICUs. The isolates were found in and around all the beds regularly occupied by patients. MLST of four CIPTO-31 isolates revealed that these could be assigned to two new STs. Another nine isolates from the largest Raman clusters could be assigned to ST195, ST208, or ST218, or a new ST based on a new allele for the *gpi* gene (http://pubmlst.org/abaumannii/). A blood culture isolate that was unique in the Raman spectroscopy typing belonged to ST642. ST195, ST208, ST218, and ST642 have all previously been identified in Asian countries [32], including China [33], Malaysia [34], and Japan [35]. The epidemiology of carbapenem-nonsusceptible *A. baumannii-calcoaceticus* complex in this Indonesian hospital was a combination of several known dominant Asian clones and new clones.

Our study has certain limitations. First, our study was a single-center study during a situation of endemic carbapenem-nonsusceptible *A. baumannii-calcoaceticus* complex colonisation and infection. Therefore, our data should not be considered to be representative for the whole country. Second, we did not evaluate the effect of other possible confounders, such as dialysis, need for inotropes, surgery, and previous admission to a hospital.

## Conclusions

In summary, this study is the largest to date that describes the characteristics and outcome of carbapenem-nonsusceptible *A. baumannii-calcoaceticus* complex in ICUs of a referral hospital in Indonesia. Colonization or infection with carbapenem-nonsusceptible *A. baumannii-calcoaceticus* complex during hospitalization was independently associated with prolonged LOS in the ICU. Prevention of *A. baumannii-calcoaceticus* complex colonization and infection requires interventions directed to source control and limiting the transmission of such strains to and between patients.

## Additional files

Additional file 1: Table S1. List of environmental samples. **Table S2.** Baseline characteristics of patients admitted to the adult and Emergency Room (ER) ICUs. **Table S3.** Variables associated with mortality among patients with and without carbapenem-nonsusceptible *A. baumannii-calcoaceticus* complex. **Table S4.** Variables associated with length of stay among patients with and without carbapenem-nonsusceptible *A. baumannii-calcoaceticus* complex. **Table S5.** Source of detection of the carbapenem-nonsusceptible *A. baumannii-calcoaceticus* complex isolates collected in the study. **Table S6.** Sources of the five major Raman clusters of carbapenem-nonsusceptible *A. baumannii-calcoaceticus* complex in adult ICU and ER-ICUs. (DOC 288 kb)

Additional file 2: Figure S1. Carbapenem-nonsusceptible *Acinetobacter baumannii-calcoaceticus* complex carriage of included patients admitted to adult and ER-ICUs of Dr. Cipto Mangunkusomo General Hospital, Jakarta, Indonesia. (TIFF 1522 kb)

Additional file 3: Figure S2. Raman spectroscopy-based cluster analysis of *Acinetobacter baumannii-calcoaceticus* complex isolates from adult and ER-ICUs. Note: Raman spectra correlation matrix of carbapenem-nonsusceptible *A. baumannii-calcoaceticus* complex isolates. Isolates are shown in a color-scale (red-orange-yellow-grey) based on their similarity of correlation coefficient value. Red clusters (91–100%) indicate isolates that are indistinguishable according to the cut-off value. Grey areas (≤70%) indicate isolates that are not related. The potentially related isolates are shown by yellow areas (lower similarities (71–80%)) and orange areas (higher similarities (81–90%)). (JPEG 761 kb)

Additional file 4: Figure S3. The bed-clone analysis of cluster CIPTO-31 carbapenem-nonsusceptible *Acinetobacter baumannii-calcoaceticus* complex. Note: The bed-clone analysis from cluster CIPTO-31 carbapenem-nonsusceptible *A. baumannii-calcoaceticus* complex showed spreading of 115 isolates in both ICUs. The isolates were found in patients from almost all the beds. A red diamond represents an environmental isolate. (TIFF 1930 kb)

## References

[1] Munoz-Price LS, Weinstein RA (2008). Acinetobacter infection. N Engl J Med.

[2] Maragakis LL, Perl TM (2008). *Acinetobacter baumannii*: epidemiology, antimicrobial resistance, and treatment options. Clin Infect Dis.

[3] Kempf M, Rolain JM (2012). Emergence of resistance to carbapenems in *Acinetobacter baumannii* in Europe: clinical impact and therapeutic options. Int J Antimicrob Agents.

[4] Centers for Disease Control and Prevention: Antibiotic resistance threats in the United States, 2013**.** Atlanta, GA: US department of health and human services, CDC 2013.

[5] Lagamayo EN (2008). Antimicrobial resistance in major pathogens of hospital-acquired pneumonia in Asian countries. Am J Infect Control.

[6] Singer M, Deutschman CS, Seymour CW, Shankar-Hari M, Annane D, Bauer M, Bellomo R, Bernard GR, Chiche JD, Coopersmith CM (2016). The third international consensus definitions for sepsis and septic shock (Sepsis-3). JAMA.

[7] The European Committee on Antimicrobial Susceptibility Testing. Breakpoint tables for interpretation of MICs and zone diameters. Version 3.1, 2013. http://www.eucast.org.

[8] Brown S, Young HK, Amyes SG (2005). Characterisation of OXA-51, a novel class D carbapenemase found in genetically unrelated clinical strains of *Acinetobacter baumannii* from Argentina. Clin Microbiol Infect.

[9] Woodford N, Ellington MJ, Coelho JM, Turton JF, Ward ME, Brown S, Amyes SG, Livermore DM, Multiplex PCR (2006). For genes encoding prevalent OXA carbapenemases in *Acinetobacter* spp. Int J Antimicrob Agents.

[10] Segal H, Garny S, Elisha BG (2005). IS IS(ABA-1) customized for *Acinetobacter*?. FEMS Microbiol Lett.

[11] Islam MA, Talukdar PK, Hoque A, Huq M, Nabi A, Ahmed D, Talukder KA, Pietroni MA, Hays JP, Cravioto A (2012). Emergence of multidrug-resistant NDM-1-producing gram-negative bacteria in Bangladesh. Eur J Clin Microbiol Infect Dis.

[12] Maquelin K, Dijkshoorn L, van der Reijden TJ, Puppels GJ (2006). Rapid epidemiological analysis of Acinetobacter strains by Raman spectroscopy. J Microbiol Methods.

[13] Willemse-Erix DF, Scholtes-Timmerman MJ, Jachtenberg JW, van Leeuwen WB, Horst-Kreft D, Bakker Schut TC, Deurenberg RH, Puppels GJ, van Belkum A, Vos MC (2009). Optical fingerprinting in bacterial epidemiology: Raman spectroscopy as a real-time typing method. J Clin Microbiol.

[14] Johnson VE (2013). Revised standards for statistical evidence. Proc Natl Acad Sci U S A.

[15] Phu VD, Wertheim HF, Larsson M, Nadjm B, Dinh QD, Nilsson LE, Rydell U, Le TT, Trinh SH, Pham HM (2016). Burden of hospital acquired infections and antimicrobial use in Vietnamese adult intensive care units. PLoS One.

[16] Apisarnthanarak A, Pinitchai U, Thongphubeth K, Yuekyen C, Warren DK, Fraser VJ, Thammasat University Pandrug-resistant Acinetobacter Baumannii control G (2008). A multifaceted intervention to reduce pandrug-resistant *Acinetobacter baumannii* colonization and infection in 3 intensive care units in a Thai tertiary care center: a 3-year study. Clin Infect Dis.

[17] Dhanoa A, Rajasekaram G, Lean SS, Cheong YM, Thong KL (2015). Endemicity of *Acinetobacter calcoaceticus-baumannii* complex in an intensive care unit in Malaysia. J Pathog.

[18] Liu Q, Li W, Du X, Li W, Zhong T, Tang Y, Feng Y, Tao C, Xie Y (2015). Risk and prognostic factors for multidrug-resistant *Acinetobacter baumannii* Complex bacteremia: a retrospective study in a tertiary Hospital of West China. PLoS One.

[19] Farida H, Severin JA, Gasem MH, Keuter M, van den Broek P, Hermans PW, Wahyono H, Verbrugh HA (2013). Nasopharyngeal carriage of *Klebsiella pneumoniae* and other gram-negative bacilli in pneumonia-prone age groups in Semarang, Indonesia. J Clin Microbiol.

[20] Latibeaudiere R, Rosa R, Laowansiri P, Arheart K, Namias N, Munoz-Price LS (2015). Surveillance cultures growing carbapenem-resistant *Acinetobacter baumannii* predict the development of clinical infections: a retrospective cohort study. Clin Infect Dis.

[21] Sunenshine RH, Wright MO, Maragakis LL, Harris AD, Song X, Hebden J, Cosgrove SE, Anderson A, Carnell J, Jernigan DB (2007). Multidrug-resistant *Acinetobacter* infection mortality rate and length of hospitalization. Emerg Infect Dis.

[22] Lemos EV, de la Hoz FP, Einarson TR, McGhan WF, Quevedo E, Castaneda C, Kawai K (2014). Carbapenem resistance and mortality in patients with *Acinetobacter baumannii* infection: systematic review and meta-analysis. Clin Microbiol Infect.

[23] Abbott I, Cerqueira GM, Bhuiyan S, Peleg AY (2013). Carbapenem resistance in *Acinetobacter baumannii:* laboratory challenges, mechanistic insights and therapeutic strategies. Expert Rev Anti-Infect Ther.

[24] Peleg AY, Seifert H, Paterson DL (2008). *Acinetobacter baumannii*: emergence of a successful pathogen. Clin Microbiol Rev.

[25] Zarrilli R, Giannouli M, Tomasone F, Triassi M. Carbapenem resistance in *Acinetobacter baumannii*: the molecular epidemic features of an emerging problem in health care facilities. J Infect Dev Ctries. 2009;3(5):335–41. 10.3855/jidc.240 19759502

[26] Rolain JM, Loucif L, Al-Maslamani M, Elmagboul E, Al-Ansari N, Taj-Aldeen S, Shaukat A, Ahmedullah H, Hamed M (2016). Emergence of multidrug-resistant *Acinetobacter baumannii* producing OXA-23 Carbapenemase in Qatar. New Microbes New Infect.

[27] Zowawi HM, Sartor AL, Sidjabat HE, Balkhy HH, Walsh TR, Al Johani SM, AlJindan RY, Alfaresi M, Ibrahim E, Al-Jardani A (2015). Molecular epidemiology of carbapenem-resistant *Acinetobacter baumannii* isolates in the Gulf cooperation council states: dominance of OXA-23-type producers. J Clin Microbiol.

[28] Nhu NTK, Lan NPH, Campbell JI, Parry CM, Thompson C, Tuyen HT, Hoang NM, Tam PTT, Le VM, Nga TVT (2014). Emergence of carbapenem-resistant *Acinetobacter baumannii* as the major cause of ventilator associated pneumonia in intensive care unit patients at an infectious disease hospital in southern Vietnam. J Med Microbiol.

[29] Phumisantiphong U, Diraphat P, Utrarachkij F, Uaratanawong S, Siripanichgon K (2009). Clonal spread of carbapenem resistant *Acinetobacter baumannii* in the patients and their environment at BMA medical college and Vajira hospital. J Med Assoc Thail.

[30] Rosa R, Depascale D, Cleary T, Fajardo-Aquino Y, Kett DH, Munoz-Price LS (2014). Differential environmental contamination with *Acinetobacter baumannii* based on the anatomic source of colonization. Am J Infect Control.

[31] Senok A, Garaween G, Raji A, Khubnani H, Kim Sing G, Shibl A (2015). Genetic relatedness of clinical and environmental *Acinetobacter baumanii* isolates from an intensive care unit outbreak. J Infect Dev Ctries.

[32] Kim DH, Choi JY, Kim HW, Kim SH, Chung DR, Peck KR, Thamlikitkul V, So TM, Yasin RM, Hsueh PR (2013). Spread of carbapenem-resistant Acinetobacter Baumannii global clone 2 in Asia and AbaR-type resistance islands. Antimicrob Agents Chemother.

[33] Qu J, Du Y, Yu R, Lu X (2016). The first outbreak caused by Acinetobacter Baumannii ST208 and ST195 in China. Biomed Res Int.

[34] Biglari S, Alfizah H, Ramliza R, Rahman MM (2015). Molecular characterization of carbapenemase and cephalosporinase genes among clinical isolates of Acinetobacter Baumannii in a tertiary medical centre in Malaysia. J Med Microbiol.

[35] Asai S, Umezawa K, Iwashita H, Ohshima T, Ohashi M, Sasaki M, Hayashi H, Matsui M, Shibayama K, Inokuchi S (2014). An outbreak of blaOXA-51-like- and blaOXA-66-positive Acinetobacter Baumannii ST208 in the emergency intensive care unit. J Med Microbiol.

