# Supplementary material for: Endemic carbapenem-nonsusceptible Acinetobacter baumannii-calcoaceticus complex in intensive care units of the national referral hospital in Jakarta, Indonesia
Source: Antimicrob Resist Infect Control. 2018 Jan 12;7:5. doi: 10.1186/s13756-017-0296-7 (PMC5767053; doi:10.1186/s13756-017-0296-7)
Supplement: Supplementary file 1 — List of environmental samples. Table S2. Baseline characteristics of patients admitted to the adult and Emergency Room (ER) ICUs. Table S3. Variables associated with mortality among patients with and without carbapenem-nonsusceptible A. baumannii-calcoaceticus complex. Table S4. Variables associated with length of stay among patients with and without carbapenem-nonsusceptible A. baumannii-calcoaceticus complex. Table S5. Source of detection of the carbapenem-nonsusceptible A. baumannii-calcoaceticus complex isolates collected in the study. Table S6. Sources of the five major Raman clusters of carbapenem-nonsusceptible A. baumannii-calcoaceticus complex in adult ICU and ER-ICUs. (DOC 288 kb) [file 13756_2017_296_MOESM1_ESM.doc]

**Supplementary Table 1. List of environmental samples**

| **Sample site** | **Number of samples** | | | |
| --- | --- | --- | --- | --- |
|  | **Adult ICU** | **ER-ICU** | | |
| Washbasin on ICUs ward | 10 | 5 | | |
| Monitor | 14 | 10 | | |
| Ventilator | 15 | 5 | | |
| Ambu bag | 8 |  | | |
| Stethoscope | 10 | 9 | | |
| Drawer handle bedside cabinet | 21 |  | | |
| Plastic multi-purpose container next to each bed | 18 | 8 | | |
| Stainless steel container | 8 | 15 | | |
| Flowmeter | 15 |  | | |
| Infusion stand | 11 | 8 | | |
| Infusion pump | 9 | 6 | | |
| Bed rails | 20 | 14 | | |
| Tap water (washbasin on ICU ward) | 10 | 8 | | |
| Chart paper on bedside cabinet | 11 | 6 | | |
| Bedside cabinet table | 15 | 11 | | |
| Cleaning room: washbasin | 9 |  | | |
| Cleaning room: sink countertop | 3 |  | | |
| Cleaning room: mug | 2 |  | | |
| Cleaning room: dish rack | 3 |  | | |
| Mattress | 5 | 4 | | |
| Comb | 3 |  | | |
| Water from siphon of washbasin | 10 | 5 | | |
| Water from mug next to each bed | 10 | 6 | | |
| Massage oil | 5 |  | | |
| Chlorine solution after use | 3 |  | |  |
| Cleaning wipes | 3 | 4 |  | |
| Wall | 3 | 2 |  | |
| Drawer of bedside cabinet | 3 |  |  | |
| Water from suction | 7 |  |  | |
| Suction connector/container | 3 |  |  | |
| Water from humidifier | 1 |  |  | |
| Water after cleaning a floor | 2 |  |  | |
| Floor | 2 |  |  | |
| Nurse station | 1 | 1 |  | |

Abbreviations: ICU,Intensive Care Unit.

**Supplementary Table 2. Baseline characteristics of patients admitted to the adult and Emergency Room (ER) ICUs**

|  | Adult ICU | ER-ICU | p value |
| --- | --- | --- | --- |
| Number of patients enrolled | 188 | 224 |  |
| Age (years), median (IQR) | 49 (38-58) | 43 (30-58) | 0.041 |
| Gender |  |  |  |
| Male (%) | 91 (48.4) | 123 (54.9) | 0.188 |
| Female (%) | 97 (51.6) | 101 (45.1) |  |
| Underlying diseases |  |  |  |
| Cardiovascular (%) | 12 (6.4) | 13 (5.8) | 0.806 |
| Cerebrovascular (%) | 10 (5.3) | 19 (8.5) | 0.211 |
| Chronic kidney disease (%) | 15 (8) | 18 (8.0) | 0.983 |
| Diabetes mellitus (%) | 80 (42.6) | 38 (17.0) | 0.000** |
| Malignancy (%) | 62 (32.9) | 120 (53.6) | 0.000** |
| Indication for ICU admission |  |  | 0.039 |
| Medical (%) | 54 (28.7) | 86 (38.4) |  |
| Surgical (%) | 134 (71.3) | 138 (61.6) |  |
| Referral from |  |  | 0.000** |
| Other ward this hospital (%) | 144 (76.6) | 78 (34.8) |  |
| Other hospital (%) | 20 (10.6) | 57 (25.4) |  |
| Directly from Emergency Unit (%) | 24 (12.8) | 89 (39.8) |  |
| Antibiotic exposure (before admission to ICU) |  |  |  |
| Any antibiotic (%) | 146 (77.7) | 165 (73.7) | 0.349 |
| Carbapenem (%) | 40 (21.3) | 39 (17.4) | 0.321 |
| SIRS Score, (%) |  |  | 0.992 |
| Score >2 | 172 (91.5) | 205 (91.5) |  |
| Score <2 | 16 (8.5) | 19 (8.5) |  |
| qSOFA Score, (%) |  |  | 0.158 |
| Score >2 | 158 (84.0) | 176 (78.6) |  |
| Score <2 | 30 (16.0) | 48 (21.4) |  |
| Procedures (during ICU admission) |  |  |  |
| Mechanical ventilation (%) | 170 (90.4) | 201 (89.7) | 0.815 |
| Mechanical ventilation (days), median (IQR) | 4 (1.5-9) | 3 (2-7) | 0.591 |
| >5 days (%) | 87 (26.3) | 95 (42.4) | 0.431 |
| <5 days (%) | 101 (53.7) | 129 (57.6) |  |
| Central venous catheter (%) | 166 (88.3) | 197 (87.9) | 0.913 |
| Central venous catheter (days), median (IQR) | 5.5 (3-10) | 5 (3-8.5) | 0.150 |
| >5 days (%) | 106 (56.4) | 117 (52.2) | 0.400 |
| <5 days (%) | 82 (43.6) | 107 (47.8) |  |
| Urine catheter (%) | 188 (100) | 224 (100) | N/A |
| Urine catheter (days), median (IQR) | 6 (3-11) | 5 (3-9) | 0.181 |
| >5 days (%) | 118 (62.8) | 133 (59.4) | 0.486 |
| <5 days (%) | 70 (37.2) | 91 (40.6) |  |
| Antibiotic therapy (during ICU admission) |  |  |  |
| Any antibiotic (%) | 188 (100) | 218 (97.3) | 0.034 |
| Carbapenem (%) | 99 (52.7) | 100 (44.6) | 0.105 |
| Outcomes |  |  |  |
| Length of stay (days), median (IQR) | 5 (3-10.75) | 5 (3-8) | 0.024 |
| Death (%) | 52 (27.7) | 67 (29.9) | 0.616 |

Abbreviations: ER-ICU, Emergency room Intensive Care Unit; ICU, Intensive Care Unit; IQR, Interquartile range; qSOFA, quick Sepsis-related Organ Failure Assessment; SIRS, Systemic Inflammatory Response Syndrome.

**p<0.01

**Supplementary Table 3. Variables associated with mortality among patients with and without** **carbapenem-nonsusceptible *A. baumannii-calcoaceticus* complex**

|  |  |  | | **Univariate analysis** | | | | | | | | **Multivariate analysis** | | | | | | | | |  |
| --- | --- | --- | --- | --- | --- | --- | --- | --- | --- | --- | --- | --- | --- | --- | --- | --- | --- | --- | --- | --- | --- |
|  | **Mortality** | | | | **p** | |  | | **99% CI** | | | | **p** | |  | | **99% CI** | |  | | |
|  | **Death** | **%** |  | | | **cOR** | | **Lower** | | **Upper** |  | | | **aOR** | | **Lower** | | **Upper** | |  | |
| Group |  |  |  | | |  | |  | |  |  | | |  | |  | |  | |  | |
| Carbapenem-NS *A. baumannii-calcoaceticus* *complex* negative | 59 | 49.6 |  | | | 1.00 | |  | |  |  | | |  | |  | |  | |  | |
| Carbapenem-NS *A. baumannii-calcoaceticus* *complex* positive on admission | 22 | 18.5 | 0.143 | | | 1.55 | | 0.72 | | 3.33 | 0.862 | | | 0.95 | | 0.41 | | 2.19 | |  | |
| Carbapenem-NS *A. baumannii-calcoaceticus* *complex* positive acquired | 38 | 31.9 | <0.01 | | | 2.46 | | 1.26 | | 4.82 | 0.119 | | | 1.59 | | 0.74 | | 3.40 | |  | |
| Gender |  |  |  | | |  | |  | |  |  | | |  | |  | |  | |  | |
| Male | 66 | 55.5 | 0.362 | | | 1.22 | | 0.70 | | 2.14 |  | | |  | |  | |  | |  | |
| Female | 53 | 44.5 |  | | | 1.00 | |  | |  |  | | |  | |  | |  | |  | |
| Underlying diseases |  |  |  | | |  | |  | |  |  | | |  | |  | |  | |  | |
| Cardiovascular |  |  |  | | |  | |  | |  |  | | |  | |  | |  | |  | |
| Yes | 9 | 7.6 | 0.418 | | | 1.42 | | 0.30 | | 4.10 |  | | |  | |  | |  | |  | |
| No | 110 | 92.4 |  | | | 1.00 | |  | |  |  | | |  | |  | |  | |  | |
| Cerebrovascular |  |  |  | | |  | |  | |  |  | | |  | |  | |  | |  | |
| Yes | 13 | 28.9 | 0.049 | | | 2.12 | | 0.78 | | 5.81 | 0.708 | | | 0.84 | | 0.26 | | 2.75 | |  | |
| No | 106 | 89.1 |  | | | 1.00 | |  | |  |  | | | 1.00 | |  | |  | |  | |
| Chronic kidney diseases |  |  |  | | |  | |  | |  |  | | |  | |  | |  | |  | |
| Yes | 11 | 9.2 | 0.556 | | | 1.25 | | 0.46 | | 3.40 |  | | |  | |  | |  | |  | |
| No | 108 | 90.8 |  | | | 1.00 | |  | |  |  | | |  | |  | |  | |  | |
| Diabetes mellitus |  |  |  | | |  | |  | |  |  | | |  | |  | |  | |  | |
| Yes | 30 | 25.4 | 0.327 | | | 0.79 | | 0.42 | | 1.48 |  | | |  | |  | |  | |  | |
| No | 89 | 74.8 |  | | | 1.00 | |  | |  |  | | |  | |  | |  | |  | |
| Malignancy |  |  |  | | |  | |  | |  |  | | |  | |  | |  | |  | |
| Yes | 43 | 36.1 | 0.036 | | | 0.63 | | 0.35 | | 1.12 | 0.294 | | | 0.75 | | 0.37 | | 1.52 | |  | |
| No | 76 | 63.9 |  | | | 1.00 | |  | |  |  | | | 1.00 | |  | |  | |  | |
| Indication for ICU admission |  |  |  | | |  | |  | |  |  | | |  | |  | |  | |  | |
| Medical | 61 | 51.3 | <0.01 | | | 2.85 | | 1.59 | | 5.10 | <0.01 | | | 1.98 | | 1.07 | | 3.68 | |  | |
| Surgical | 58 | 48.7 |  | | | 1.00 | |  | |  |  | | | 1.00 | |  | |  | |  | |
| Referral from |  |  |  | | |  | |  | |  |  | | |  | |  | |  | |  | |
| Other ward this hospital | 58 | 48.7 | 0.798 | | | 1.69 | | 0.75 | | 3.80 | 0.171 | | | 1.53 | | 0.67 | | 3.50 | |  | |
| Other hospital | 30 | 25.2 | 0.096 | | | 0.94 | | 0.48 | | 1.83 | 0.182 | | | 1.50 | | 0.70 | | 3.19 | |  | |
| Directly from Emergency Unit | 31 | 26.1 |  | | | 1.00 | |  | |  |  | | | 1.00 | |  | |  | |  | |
| Antibiotic exposure (before admission to ICU) |  |  |  | | |  | |  | |  |  | | |  | |  | |  | |  | |
| Any antibiotic | 100 | 84.0 | 0.011 | | | 2.05 | | 0.99 | | 4.23 | 0.264 | | | 1.44 | | 0.62 | | 3.31 | |  | |
| No any antibiotic | 19 | 16.0 |  | | | 1.00 | |  | |  |  | | | 1.00 | |  | |  | |  | |
| Carbapenem | 36 | 30.3 | <0.01 | | | 2.52 | | 1.29 | | 4.91 | 0.111 | | | 1.61 | | 0.75 | | 3.46 | |  | |
| No carbapenem | 83 | 69.7 |  | | | 1.00 | |  | |  |  | | | 1.00 | |  | |  | |  | |
| SIRS score |  |  |  | | |  | |  | |  |  | | |  | |  | |  | |  | |
| Score ≥2 | 112 | 94.1 |  | | | 1.00 | |  | |  |  | | |  | |  | |  | |  | |
| Score <2 | 7 | 5.9 | 0.230 | | | 1.69 | | 0.55 | | 5.22 |  | | |  | |  | |  | |  | |
| Q Sofa |  |  |  | | |  | |  | |  |  | | |  | |  | |  | |  | |
| Score ≥2 | 101 | 84.9 |  | | | 1.00 | |  | |  |  | | |  | |  | |  | |  | |
| Score <2 | 18 | 15.1 | 0.211 | | | 1.45 | | 0.68 | | 3.08 |  | | |  | |  | |  | |  | |
| Procedures (during ICU admission) |  |  |  | | |  | |  | |  |  | | |  | |  | |  | |  | |
| Mechanical ventilation used (%) | 119 | 100.0 | N/A | | |  | |  | |  |  | | |  | |  | |  | |  | |
| No mechanical ventilation used (%) | 0 | 0.0 |  | | |  | |  | |  |  | | |  | |  | |  | |  | |
| Mechanical Ventilation (days) |  |  |  | | |  | |  | |  |  | | |  | |  | |  | |  | |
| >5 days | 74 | 62.2 | <0.01 | | | 2.82 | | 1.58 | | 5.02 | <0.01 | | | 4.71 | | 1.36 | | 16.30 | |  | |
| <5 days | 45 | 37.8 |  | | | 1.00 | |  | |  |  | | | 1.00 | |  | |  | |  | |
| Central venous catheter use | 116 | 97.5 | <0.01 | | | 7.20 | | 1.51 | | 34.34 | <0.01 | | | 5.76 | | 1.06 | | 31.20 | |  | |
| No central venous catheter used | 3 | 2.5 |  | | | 1.00 | |  | |  |  | | | 1.00 | |  | |  | |  | |
| Central Venous Catheter (days) |  |  |  | | |  | |  | |  |  | | |  | |  | |  | |  | |
| >5 days | 77 | 64.7 | 0.006 | | | 1.85 | | 1.04 | | 3.29 | 0.011 | | | 3.59 | | 0.99 | | 13.05 | |  | |
| <5 days | 42 | 35.3 |  | | | 1.00 | |  | |  |  | | | 1.00 | |  | |  | |  | |
| Urine catheter used | 119 | 100.0 | N/A | | |  | |  | |  |  | | |  | |  | |  | |  | |
| No urine catheter used | 0 | 0.0 |  | | |  | |  | |  |  | | |  | |  | |  | |  | |
| Urine Catheter (days) |  |  |  | | |  | |  | |  |  | | |  | |  | |  | |  | |
| >5 days | 85 | 71.4 | 0.005 | | | 1.91 | | 1.05 | | 3.50 | 0.647 | | | 1.29 | | 0.31 | | 5.26 | |  | |
| <5 days | 34 | 28.6 |  | | | 1.00 | |  | |  |  | | | 1.00 | |  | |  | |  | |
| Antibiotic therapy (during ICU admission) |  |  |  | | |  | |  | |  |  | | |  | |  | |  | |  | |
| Any antibiotic | 119 | 100.0 | N/A | | |  | |  | |  |  | | |  | |  | |  | |  | |
| No any antibiotic | 0 | 0.0 |  | | | 1.00 | |  | |  |  | | |  | |  | |  | |  | |
| Carbapenem | 81 | 68.1 | <0.01 | | | 3.16 | | 1.75 | | 5.72 | 0.002 | | | 2.17 | | 1.13 | | 4.16 | |  | |
| No carbapenem | 38 | 31.9 |  | | | 1.00 | |  | |  |  | | | 1.00 | |  | |  | |  | |
| Age, median (IQR) (years old) | 50 | (38-60) | 0.009 | | | 1.02 | | 1.00 | | 1.04 | 0.166 | | | 1.01 | | 0.99 | | 1.03 | |  | |
| LOS, median (IQR) (days) | 6 | (3-12) | 0.058 | | | 1.03 | | 0.99 | | 1.06 |  | | |  | |  | |  | |  | |

Abbreviation: aOR, adjusted Odds Ratio; cOR, crude Odds Ratio; CI, Confidence Interval; ICU: Intensive Care Unit; IQR, Interquartile range; LOS, Length of Stay; NS, Nonsusceptible; SIRS, Systemic Inflammatory Response Syndrome; qSOFA, quick Sepsis-related Organ Failure Assessment.

**Supplementary Table 4. Variables associated with length of stay among patients with and without carbapenem-nonsusceptible *A. baumannii-calcoaceticus* complex**

| **Variable** |  | **Univariate analysis** | | | | **Multivariate analysis** | | | | |
| --- | --- | --- | --- | --- | --- | --- | --- | --- | --- | --- |
|  | **Length of stay** | **p** | **cHR** | **99% CI** | | | **p** | **aHR** | **99% CI** | |
|  | **Median (IQR)** |  |  | **Lower** | **Upper** | |  |  | **Lower** | **Upper** |
| Group |  |  |  |  |  | |  |  |  |  |
| Carbapenem-NS *A. baumannii-calcoaceticus* *complex* negative | 4 (3-7) |  | 1.00 |  |  | |  | 1.00 |  |  |
| Carbapenem-NS *A. baumannii-calcoaceticus* *complex* positive on admission | 5 (3-9) | 0.045 | 1.31 | 0.93 | 1.87 | | 0.755 | 1.04 | 0.73 | 1.50 |
| Carbapenem-NS *A. baumannii-calcoaceticus* *complex* positive, acquired | 11 (5-18) | <0.01 | 2.73 | 1.93 | 3.88 | | <0.01 | 2.56 | 1.76 | 3.70 |
| Gender |  |  |  |  |  | |  |  |  |  |
| Male | 5 (3-10) | 0.426 | 0.92 | 0.72 | 1.19 | |  |  |  |  |
| Female | 4 (3-9) |  | 1.00 |  |  | |  |  |  |  |
| Underlying disease |  |  |  |  |  | |  |  |  |  |
| Cardiovascular |  |  |  |  |  | |  |  |  |  |
| Yes | 8 (5-12) | 0.199 | 1.31 | 0.77 | 2.22 | |  |  |  |  |
| No | 5 (3-9) |  | 1.00 |  |  | |  |  |  |  |
| Cerebrovascular |  |  |  |  |  | |  |  |  |  |
| Yes | 8 (3-14) | 0.026 | 1.54 | 0.93 | 2.55 | | 0.580 | 0.89 | 0.52 | 1.53 |
| No | 5 (3-9) |  | 1.00 |  |  | |  | 1.00 |  |  |
| Chronic kidney disease |  |  |  |  |  | |  |  |  |  |
| Yes | 7 (4-14) | 0.108 | 1.34 | 0.84 | 2.14 | |  |  |  |  |
| No | 5 (3-9) |  | 1.00 |  |  | |  |  |  |  |
| Diabetes mellitus |  |  |  |  |  | |  |  |  |  |
| Yes | 4 (3-9) | 0.214 | 0.87 | 0.66 | 1.16 | |  |  |  |  |
| No | 5 (3-9) |  | 1.00 |  |  | |  |  |  |  |
| Malignancy |  |  |  |  |  | |  |  |  |  |
| Yes | 5 (3-8) | 0.254 | 0.89 | 0.69 | 1.15 | |  |  |  |  |
| No | 5 (3-10) |  | 1.00 |  |  | |  |  |  |  |
| Indication for ICU admission |  |  |  |  |  | |  |  |  |  |
| Medical | 6 (3-12) | <0.01 | 1.52 | 1.15 | 2.01 | | 0.599 | 1.06 | 0.79 | 1.44 |
| Surgical | 4 (3-8) |  | 1.00 |  |  | |  | 1.00 |  |  |
| Referral from |  |  |  |  |  | |  |  |  |  |
| Other ward this hospital | 5 (3-10) | 0.151 | 1.18 | 0.88 | 1.59 | |  |  |  |  |
| Other hospital | 5 (3-9) | 0.197 | 1.21 | 0.83 | 1.77 | |  |  |  |  |
| Directly from Emergency Unit | 5 (3-8) |  | 1.00 |  |  | |  |  |  |  |
| Antibiotic exposure (before admission to ICU) |  |  |  |  |  | |  |  |  |  |
| Any antibiotic | 5 (3-10) | 0.014 | 1.33 | 0.99 | 1.79 | | 0.037 | 1.28 | 0.94 | 1.74 |
| No antibiotic | 4 (3-7) |  | 1.00 |  |  | |  | 1.00 |  |  |
| Carbapenem | 8 (3-13) | 0.012 | 1.37 | 0.99 | 1.90 | | 0.168 | 1.22 | 0.84 | 1.75 |
| No carbapenem | 5 (3-8) |  | 1.00 |  |  | |  | 1.00 |  |  |
| SIRS score |  |  |  |  |  | |  |  |  |  |
| Score >2 | 5 (3-9) | 0.651 | 1.08 | 0.69 | 1.71 | | 0.255 | 1.23 | 0.77 | 1.99 |
| Score <2 | 4 (2-7) |  | 1.00 |  |  | |  | 1.00 |  |  |
| qSOFA |  |  |  |  |  | |  |  |  |  |
| Score >2 | 5 (3-10) | <0.01 | 1.62 | 1.17 | 2.25 | | 0.112 | 1.24 | 0.88 | 1.74 |
| Score <2 | 3 (2-6) |  | 1.00 |  |  | |  | 1.00 |  |  |
| Procedures (during ICU admission) |  |  |  |  |  | |  |  |  |  |
| Mechanical ventilation | 5 (3-10) | <0.01 | 2.50 | 1.61 | 3.89 | | 0.659 | 1.09 | 0.67 | 1.77 |
| No mechanical ventilation | 3 (2-4) |  | 1.00 |  |  | |  | 1.00 |  |  |
| Mechanical Ventilation (days) |  |  |  |  |  | |  |  |  |  |
| >5 days | 10 (7-15) | <0.01 | 6.33 | 4.592 | 8.727 | | <0.01 | 3.10 | 2.00 | 4.79 |
| <5 days | 3 (2-4) |  | 1.00 |  |  | |  | 1.00 |  |  |
| Central venous catheter | 5 (3-10) | <0.01 | 2.12 | 1.42 | 3.17 | | 0.462 | 0.87 | 0.53 | 1.43 |
| No central venous catheter | 3 (2-5) |  | 1.00 |  |  | |  | 1.00 |  |  |
| Central venous catheter (days) |  |  |  |  |  | |  |  |  |  |
| >5 days | 9 (6-13) | <0.01 | 6.30 | 4.62 | 8.59 | | 0.009 | 1.77 | 1.01 | 3.08 |
| <5 days | 3 (2-3) |  | 1.00 |  |  | |  | 1.00 |  |  |
| Urine catheter | 5 (3-9) | N/A |  |  |  | |  |  |  |  |
| No urine catheter | N/A |  |  |  |  | |  |  |  |  |
| Urine catheter (days) |  |  |  |  |  | |  |  |  |  |
| >5 days | 8 (5-12) | <0.01 | 9.87 | 6.84 | 14.24 | | <0.01 | 3.26 | 1.85 | 5.74 |
| <5 days | 3 (2-3) |  | 1.00 |  |  | |  | 1.00 |  |  |
| Antibiotic therapy (during ICU admission) |  |  |  |  |  | |  |  |  |  |
| Any antibiotic | 5 (3-9) | 0.005 | 3.20 | 1.10 | 9.33 | | 0.344 | 1.49 | 0.50 | 4.43 |
| No antibiotic | 2 (2-3) |  | 1.00 |  |  | |  | 1.00 |  |  |
| Carbapenem | 7 (4-12) | <0.01 | 1.78 | 1.37 | 2.32 | | 0.570 | 0.93 | 0.68 | 1.28 |
| No carbapenem | 4 (2-7) |  |  |  |  | |  | 1.00 |  |  |
| Age, correlation coefficient (r) | 0.081 | 0.154 | 1.00 | 0.99 | 1.00 | |  |  |  |  |
| Mortality | 6 (3-12) | 0.057 | 1.23 | 0.93 | 1.63 | | 0.016 | 0.76 | 0.57 | 1.02 |

Abbreviation: aHR, adjusted Hazard Ratio; cHR, crude Hazard Ratio; CI, Confidence Interval, ICU: Intensive Care Unit; IQR, Interquartile Range; LOS, Length of Stay; NS, Nonsusceptible; SIRS, Systemic Inflammatory Response Syndrome; qSOFA, quick Sepsis-related Organ Failure Assessment .

**Supplementary Table 5. Source of detection of the carbapenem-nonsusceptible *A. baumannii-calcoaceticus* complex isolates collected in the study**

| **Culture** | **Number of patients** | **Isolates** |
| --- | --- | --- |
| Only screening | 80 | 131 |
| Only clinical specimen | 34 | 49 |
| Screening and clinical specimen | 44 | 131 |
| Environment |  | 6 |
| Healthcare worker screening |  | 1 |
| **Total** | **158** | **318** |

**Supplementary Table 6. Sources of the five major Raman clusters of carbapenem-nonsusceptible *A. baumannii-calcoaceticus* complex in adult ICU and ER-ICUs**

| Raman  cluster | Number of isolates (patients) | Origin of strain | | | | | |
| --- | --- | --- | --- | --- | --- | --- | --- |
|  |  | Patients | | | | Environment | |
|  |  | Adult ICU |  | ER-ICU |  | Adult ICU | ER-ICU |
|  |  | Screening | Clinical | Screening | Clinical |  |  |
| CIPTO-30 | 23 (14) | 12 | 6 | 4 | 1 |  |  |
| CIPTO-31 | 111 (69) | 35 | 17 | 33 | 26 | 4 |  |
| CIPTO-45 | 33 (19) | 8 | 4 | 17 | 4 |  |  |
| CIPTO-46 | 36 (27) | 7 | 10 | 10 | 9 |  |  |
| CIPTO-48 | 40 (29) | 12 | 9 | 14 | 5 | 1 |  |

Abbreviation: ER-ICU, Emergency room Intensive Care Unit; ICU, Intensive Care Unit.

Five largest Raman clusters are indicated by CIPTO-30, CIPTO-31, CIPTO-45, CIPTO-46, and CIPTO-48.
