# Supplementary figures and images for: Endemic carbapenem-nonsusceptible Acinetobacter baumannii-calcoaceticus complex in intensive care units of the national referral hospital in Jakarta, Indonesia
Source: Antimicrob Resist Infect Control. 2018 Jan 12;7:5. doi: 10.1186/s13756-017-0296-7 (PMC5767053; doi:10.1186/s13756-017-0296-7)

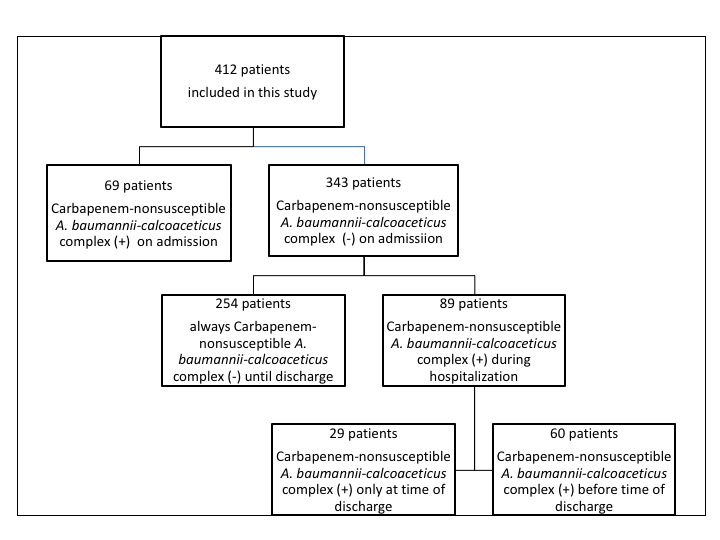

Supplement: Supplementary file 2 — Carbapenem-nonsusceptible Acinetobacter baumannii-calcoaceticus complex carriage of included patients admitted to adult and ER-ICUs of Dr. Cipto Mangunkusomo General Hospital, Jakarta, Indonesia. (TIFF 1522 kb) [file 13756_2017_296_MOESM2_ESM.tiff]

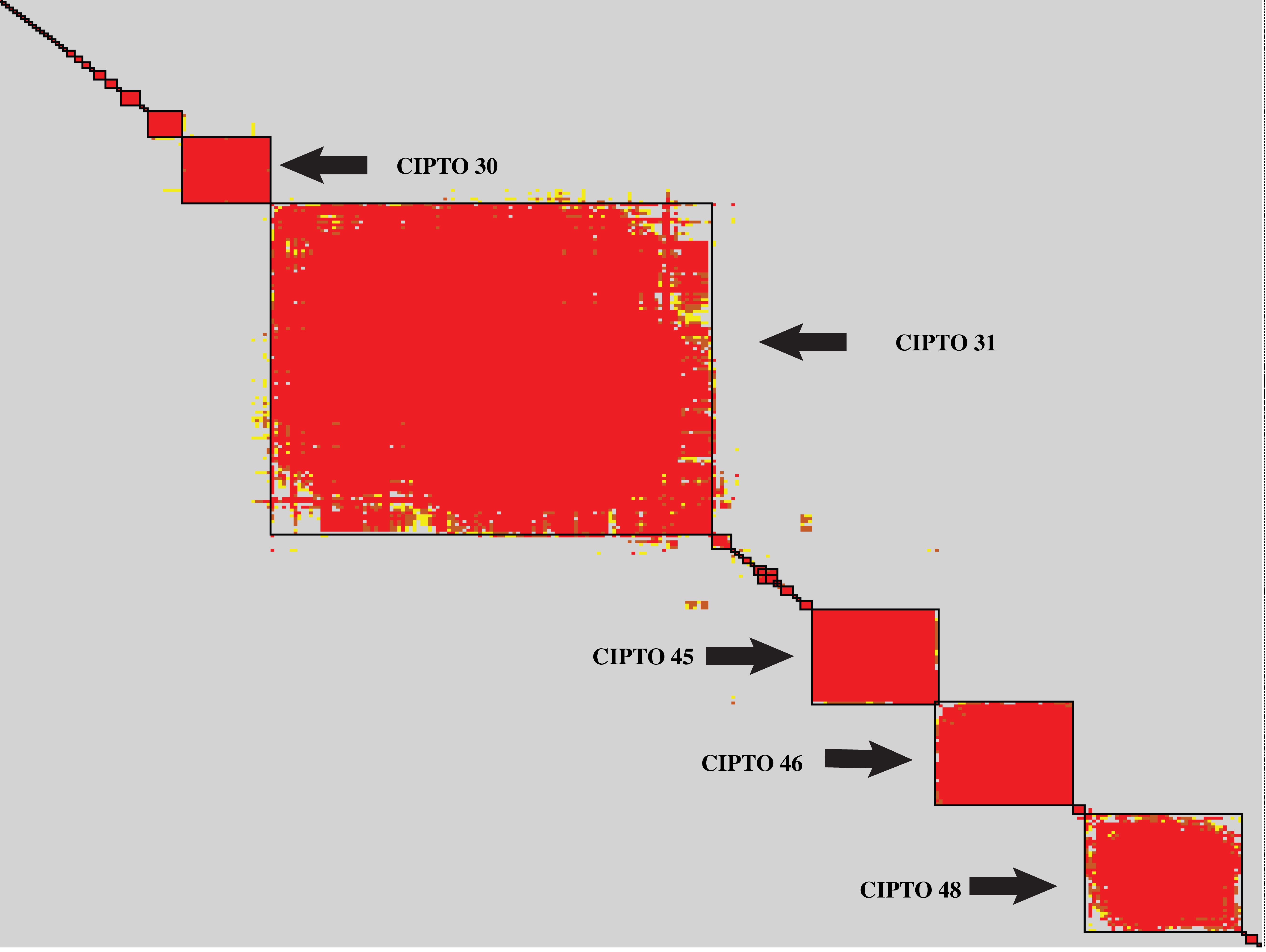

Supplement: Supplementary file 3 — Raman spectroscopy-based cluster analysis of Acinetobacter baumannii-calcoaceticus complex isolates from adult and ER-ICUs. Note: Raman spectra correlation matrix of carbapenem-nonsusceptible A. baumannii-calcoaceticus complex isolates. Isolates are shown in a color-scale (red-orange-yellow-grey) based on their similarity of correlation coefficient value. Red clusters (91–100%) indicate isolates that are indistinguishable according to the cut-off value. Grey areas (≤70%) indicate isolates that are not related. The potentially related isolates are shown by yellow areas (lower similarities (71–80%)) and orange areas (higher similarities (81–90%)). (JPEG 761 kb) [file 13756_2017_296_MOESM3_ESM.jpg]

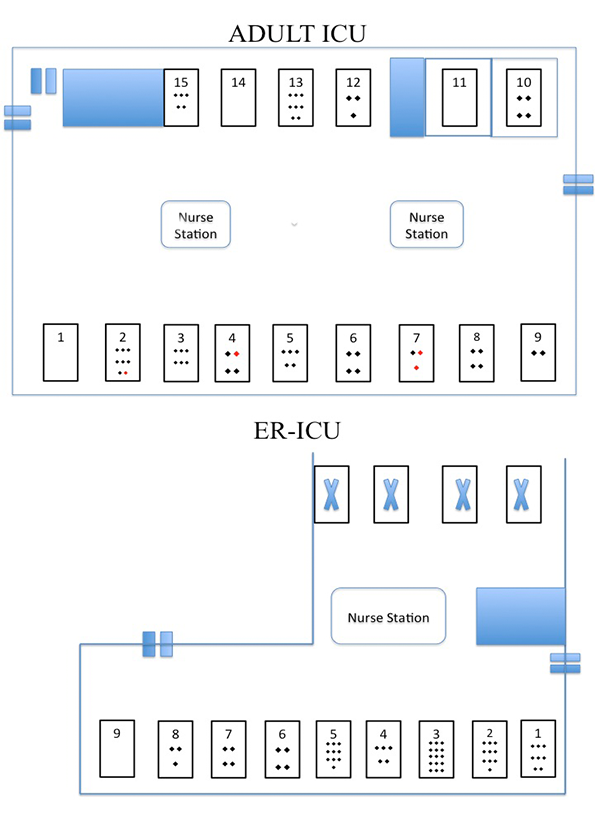

Supplement: Supplementary file 4 — The bed-clone analysis of cluster CIPTO-31 carbapenem-nonsusceptible Acinetobacter baumannii-calcoaceticus complex. Note: The bed-clone analysis from cluster CIPTO-31 carbapenem-nonsusceptible A. baumannii-calcoaceticus complex showed spreading of 115 isolates in both ICUs. The isolates were found in patients from almost all the beds. A red diamond represents an environmental isolate. (TIFF 1930 kb) [file 13756_2017_296_MOESM4_ESM.tif]
